# Comparative outcomes of retroperitoneal partial nephrectomy for cT1 and cT2 renal tumors: a single-center experience

**DOI:** 10.1186/s12894-025-01786-8

**Published:** 2025-05-02

**Authors:** Ren-Jie Lin, Chia-Chih Hsieh, Wen-Hsin Tseng, Chien-Liang Liu, Steven K. Huang, Allen W. Chiu

**Affiliations:** 1 Department of General Medicine, Chi Mei Medical Center, Tainan, Taiwan; 2 Division of Urology, Department of Surgery, Chi Mei Medical Center, Tainan, Taiwan; 3 Division of Uro-Oncology, Department of Surgery, Chi Mei Medical Center, Tainan, Taiwan; 4 Institute of Biomedical Science, National Sun Yat-Sen University, Kaohsiung, Taiwan; 5 Department of Urology, Shin Kong Wu Ho-Su Memorial Hospital, Taipei, Taiwan

**Keywords:** Large renal tumor, Partial nephrectomy, Retroperitoneal approach

## Abstract

**Background:**

Partial nephrectomy (PN) has been the main strategy for treating cT1 (≤ 7 cm) renal tumors. Previous studies have established PN’s safety and effectiveness over radical nephrectomy (RN) for cT1 tumors. However, the efficacy and safety of retroperitoneal PN for larger renal tumors (> 7 cm) remained controversial. Through a size-based comparative analysis of cT1 and cT2 tumors undergoing retroperitoneal PN, we explored the impact of renal tumors larger than 7 cm on perioperative, oncological, and functional outcomes.

**Materials and methods:**

From January 2017 to April 2021, we collected data from 201 patients undergoing retroperitoneal laparoscopic or robot-assisted PN. Of these, 173 (86.1%) had tumors ≤ 7 cm (Group A) and 28 (13.9%) had tumors > 7 cm (Group B). We analyzed demographics (gender, age, Body Mass Index, Charlson Comorbidity Index, preoperative hemoglobin and renal function, tumor location, operative method, RENAL score, and complexity), perioperative (operative time, warm ischemic time, estimated blood loss, hospital stay, surgical margins, complications), and functional outcomes (changes in renal function pre- and postoperatively), along with recurrence rates.

**Results:**

Mean tumor sizes in Group A and Group B were 3.67 ± 1.56 cm and 9.90 ± 2.97 cm, respectively. RENAL score analysis revealed a significant difference (7.64 vs. 9.21, *P* < 0.0001), attributed to the Radius and Exophytic/Endophytic property parameters. Furthermore, Group B exhibited significantly higher tumor complexity(*P* = 0.0009). In perioperative outcomes, Group B had a prolonged warm ischemic time (18.90 vs. 22.60 min, *P* = 0.0486). However, there was no significant difference in estimated blood loss and complication rates. Regarding functional outcomes, only the reduction of estimated glomerular filtration rate on postoperative day 1 was significant (-0.74 vs. -8.31, *p* = 0.016), with no significant differences at 3 months, 6 months, or 1 year postoperatively. For eGFR changes over time in Group B, declines at postoperative month 3 and postoperative year 1 were noted.

**Conclusion:**

Despite higher preoperative RENAL scores and prolonged perioperative warm ischemic time, retroperitoneal PN for tumors > 7 cm demonstrated acceptable functional, oncological, and perioperative outcomes, with no observed gastrointestinal complications. Our findings support its feasibility as a treatment option for patients with > 7 cm or intermediate/high complexity renal tumors.

## Background

In the treatment of renal tumors, the safety and efficacy of partial nephrectomy (PN) for cT1 tumors (≤ 7 cm) have been demonstrated in previous studies, and PN has been considered one of the standard treatments in the past decades. Initially, PN was limited to tumors ≤ 4 cm, with poorer survival outcomes noted when the tumor size was beyond 4 cm [1, 2]. 

However, accumulating evidence has extended its indications to larger tumors. Studies have shown that for cT1b tumors (4–7 cm), PN provides equivalent cancer-specific survival to RN while significantly reducing renal function impairment [3–5]. 

Despite these advancements, the role of PN in tumors > 7 cm (cT2) remains controversial due to concerns regarding surgical complexity, perioperative complications, and oncological outcomes. A recent systematic review highlighted the feasibility of RAPN (robotic-assisted partial nephrectomy) for selected cT2 tumors. Minimally invasive techniques, including RAPN and LPN (laparoscopic partial nephrectomy), have expanded the indication of nephron-sparing surgery beyond traditional limitations [6]. Simultaneously, less invasive treatment strategies are increasingly gaining attention in the management of renal tumors, such as percutaneous cryoablation as an alternative treatment for cT1a tumors. A recent study comparing RAPN and cryoablation demonstrated that while cryoablation was associated with a lower complication rate and shorter hospital stay, it may carry a higher risk of recurrence [7]. Therefore, RAPN or LPN remains the preferred approach for long-term oncological control in the treatment of localized renal masses.

The efficacy and safety of retroperitoneal PN for renal tumors larger than 7 cm are currently unclear. Despite existing evidence, data on the outcomes of PN performed via the retroperitoneal approach in the treatment of renal tumors, including cT2 or high-complexity tumors, are relatively scarce. Thus, through a size-based comparative analysis of cT1 and cT2 tumors, we aim to evaluate the functional, perioperative, and oncological outcomes of retroperitoneal PN for renal tumors larger than 7 cm.

## Materials and methods

### Patients and procedures

From January 2017 to April 2021, 201 patients diagnosed with localized renal cell carcinoma at Chimei Medical Center were retrospectively reviewed. All patients underwent retroperitoneal laparoscopic or robot-assisted PN. The inclusion criteria included: patients diagnosed with cT1 ~ cT2 RCC based on computed tomography (CT) findings, aged > 18 years, and complete follow-up data available. The choice of surgical approach, either laparoscopic or robotic-assisted, was determined by patient preference or economic consideration after thorough explanation of the risks associated with each method. The exclusion criteria included those diagnosed with advanced RCC on CT, encompassing extensive tumor invasion into renal vein, inferior vena cava, or beyond Gerota’s fascia (cT3 ~ cT4), the presence of distant metastasis, or those with incomplete follow-up data. These patients were excluded from our cohort in the early stage of data collection. During the follow-up period, no mortality or loss of follow-up occurred among our patients. The allocation was decided based on the tumor size measured on preoperative CT, with a cutoff of 7 cm. 173 (86.1%) had tumors ≤ 7 cm (Group A) and 28 (13.9%) had tumors > 7 cm (Group B). The description of TNM stage for tumors was according to AJCC 8th edition [8]. The achievement of trifecta must fulfill all of the following criteria: negative surgical margin, no Clavien-Dindo classification system (CDS) > 3 complication, and postoperative eGFR (estimated glomerular filtration rate) decline < 30%.

### Collection of clinical data

We retrospectively reviewed medical records of 201 patients who underwent a retroperitoneal approach of partial nephrectomy via either laparoscopic or robotic-assisted method. All patients were followed up for at least 12 months with well-documented datas. The impact of partial nephrectomy on renal function was assessed by measuring reductions in postoperative eGFR at different time points: day 1 (POD1), 3 months (POM3), 6 months (POM6), and 1 year (POY1). All eGFR values were calculated using the Modification of Diet in Renal Disease (MDRD) equation. Complications were classified according to the CDS.

### Surgical procedure

**Fig. 1:**
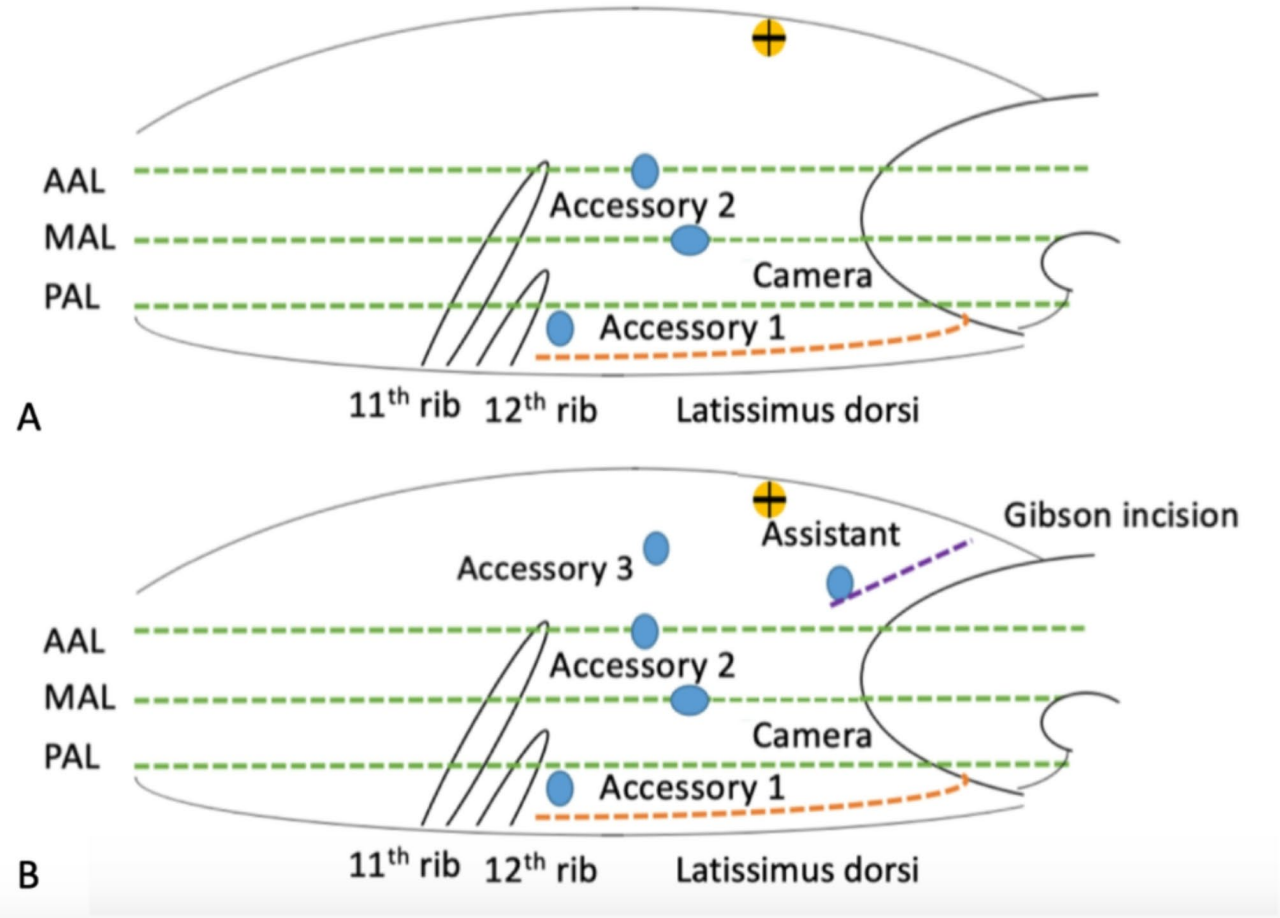
(**A**) Trocar settings for laparoscopic partial nephrectomy; (**B**) Trocar settings for robotic-assisted laparoscopic partial nephrectomy

All of the PN in this study were contributed to 3 surgeons in our institute. They all experienced complete training course of Urology residency at Chimei medical center. Besides, they had more than 10 years surgical experience on laparoscopic technique. In LPN, the patients were placed in the full flank position with the ipsilateral side facing up. The operation table was flexed to expand the working space between the 12th rib and the iliac crest. Skin preparation was conducted from the xiphoid process to pubic symphysis in the abdomen, and from the ipsilateral paraspinal region posteriorly to the umbilicus anteriorly. An approximate 2 cm incision was made above the iliac crest along the middle axillary line for the camera port. Two accessory working ports were inserted under endoscopic monitoring approximately 7 cm away from the camera port in the anterior axillary line and the corner between 12th rib and Latissimus dorsi muscle. In RAPN, additional accessory working ports (accessory 3) were placed approximately 7 cm away from the accessory-2 port. Assistant port was placed at the top of the Gibson incision line. (Figure 1A and B)

First, the laparoscopic examination revealed the psoas muscle & ureter clearly before identifying and exposing the pedicle of the selected kidney. The renal artery and the tumor were identified using a 10 mm, 0-degree laparoscope after nephrolysis. An intra-abdominal ultrasound was used to guide the tumor margin and depth of penetration. Then the renal tumor was resected after renal artery controlled with the Bulldog clip. After achieving hemostasis, a double-layer renorrhaphy was performed. A 5 mm closed wound vacuum (CWV) drain was left, which was removed when the drainage volume decreased to < 50 ml/day.

**Fig. 2:**
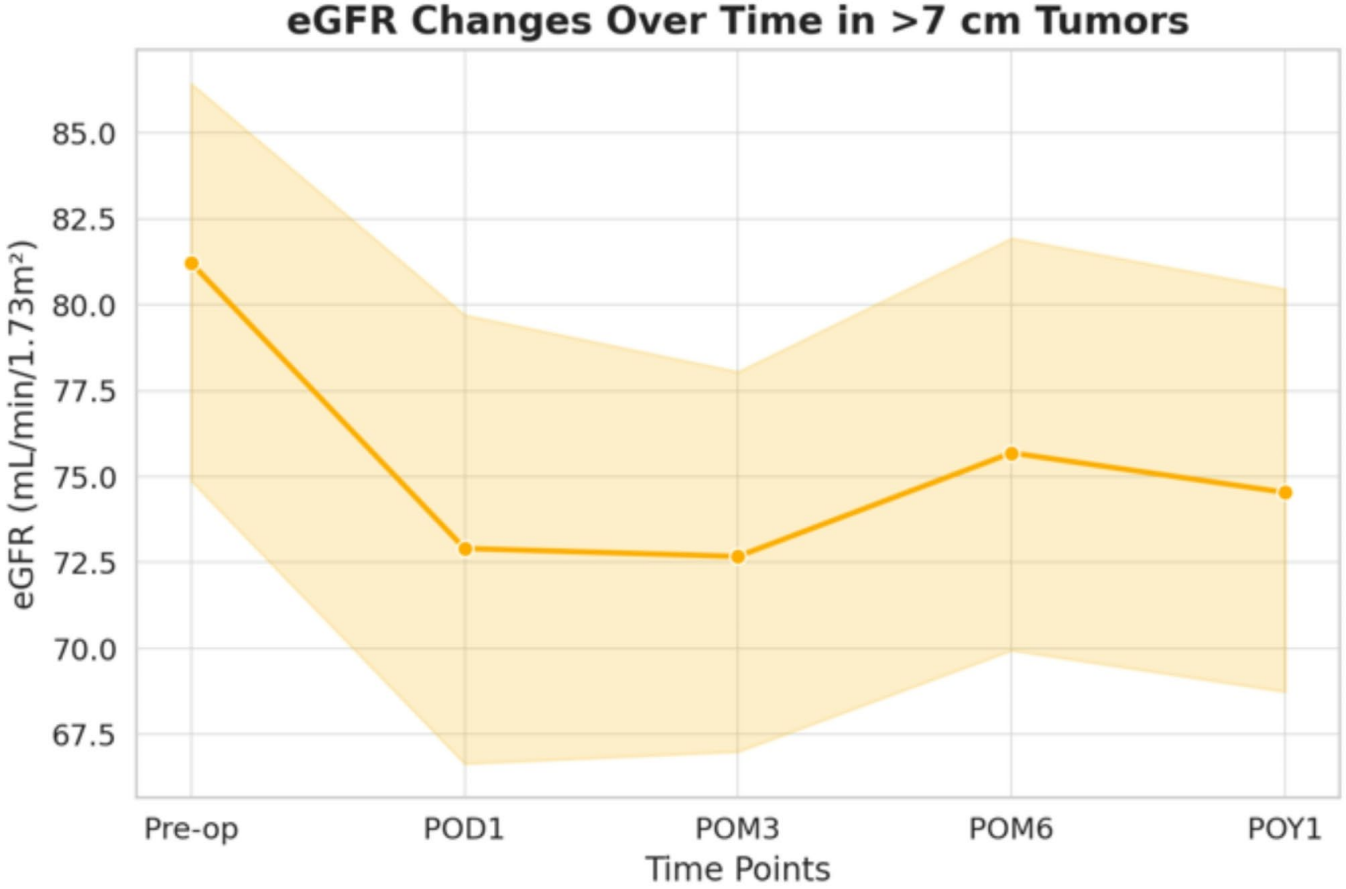
eGFR Changes Over Time in > 7 cm Tumors

### Statistical analysis

Patient characteristics and surgical outcomes were depicted using descriptive statistics. Continuous variables were presented as mean ± standard deviation, while categorical variables were expressed as frequencies (percentages). To assess the distribution difference between the Group A(≤ 7 cm) and Group B(> 7 cm), categorical variables were analyzed by the Chi-Squared Test or Fisher’s exact test while continuous variables were analyzed by the independent sample t-test. Repeated measures ANOVA (Analysis of variance) was used to assess changes in estimated glomerular filtration rate (eGFR) over time. Mauchly’s test of sphericity was conducted to evaluate the assumption of sphericity. If the assumption was violated (*p* < 0.05), the Greenhouse-Geisser correction was applied to adjust the degrees of freedom. Pairwise comparisons were conducted using Bonferroni correction for multiple testing. All of the statistical data was analyzed via IBM SPSS Statistics Version 27. In all statistical analysis, *P* < 0.05 was used to indicate statistical significance.

### Ethic approval

This study was approved by the Institutional Review Board Committee [Chimei Hospital Institutional Review Board (IRB) number 11306–001] and the Chi Mei Hospital IRB waived requirements for informed consent.

## Results

The demographic and characteristics of the renal tumor are presented in Table 1. Out of 201 patients with renal tumors receiving PN, 173 (86.1%) had tumors ≤ 7 cm (cT1, Group A) and 28 (13.9%) had tumors > 7 cm (cT2, Group B). The mean tumor size in Group A and Group B are 3.67 ± 1.56 cm and 9.90 ± 2.97 cm, respectively. Among them, 100 patients were treated with LPN while the other 101 patients were treated with RAPN, all via retroperitoneal approach. There is no difference in gender, age group, body mass index (BMI), Charlson Comorbidity Index(CCI), preoperative hemoglobin and eGFR, and tumor location between both groups. However, a significantly higher RENAL score in Group B was observed (7.64 vs. 9.2, *P* < 0.0001), resulting from parameters of Radius and Exophytic/Endophytic property. Likewise, Group B also exhibited a higher tumor complexity (Low: 75(43.35%) vs. 33(10.71%), Intermedium: 74(42.77%) vs. 16(57.14%), and High: 24(13.87%) vs. 9(32.14%), respectively, *P* = 0.0009).

**Table 1:** Demographic information of patient with renal tumor

	Overall	Tumor size ≤ 7 cm	Tumor size > 7 cm	*P* value*
Number of patients, n	201	173	28	
Gender, n(%)				0.4711
Female	95(47.26)	80(46.24)	15(53.57)	
Male	106(52.74)	93(53.76)	13(46.43)	
Age group, n(%)				0.1461
<65	145	128	17	
≧65	56	45	11	
BMI (kg/m2, mean ± SD)	25.27 ± 4.20	25.44 ± 4.33	24.26 ± 3.12	0.1675
CCI index (mean ± SD)	2.28 ± 1.87	2.25 ± 1.91	2.5 ± 1.64	0.5110
Preoperative Hb (g/dL, mean ± SD)	13.42 ± 1.79	13.49 ± 1.87	13.00 ± 1.10	0.0590
Preoperative eGFR (mean ± SD)	79.37 ± 18.06	79.07 ± 18.50	81.22 ± 15.30	0.5598
Mean follow-up (months ± SD)	41.87 ± 17.53	40.82 ± 16.52	48.35 ± 22.07	0.0934
Tumor location, n(%)				0.3681
Left	99(49.25)	83	16	
Right	102(50.75)	90	12	
HTN, yes	96(47.76)	81(46.82)	15(53.57)	0.5070
DM, yes	50(24.88)	41(23.70)	9(32.14)	0.3376
Operation method, n(%)				0.4316
Laparoscopic	100	88(50.87)	12(42.86)	
Robotic-assisted	101	85(49.13)	16(57.14)	
Tumor size, mean(SD)	4.54(2.82)	3.67(1.56)	9.90(2.97)	< 0.00001
R, n(%)				< 0.0001
1	117	117(67.63)	0(0.00)	
2	47	47(27.17)	0(0.00)	
3	37	9(5.20)	28(100.00)	
E, n(%)				0.0029
1	99	78(45.09)	21(75.00)	
2	72	65(37.57)	7(25.00)	
3	30	30(17.34)	0(0.00)	
N, n(%)				0.8059
1	80	70(40.46)	10(35.71)	
2	32	28(16.18)	4(14.29)	
3	89	75(43.35)	14(50.00)	
A, n(%)				0.4578
Posterior	92	81(46.82)	11(39.29)	
Anterior	109	92(53.18)	17(60.71)	
L, n(%)				0.3937
1	69	62(35.84)	7(25.00)	
2	60	52(30.06)	8(28.57)	
3	72	59(34.10)	13(46.43)	
RENAL score	7.86 ± 2.19	7.64 ± 2.20	9.21 ± 1.62	< 0.0001
Complexity, n(%)				0.0009
Low	78	75(43.35)	3(10.71)	
Intermediate	90	74(42.77)	16(57.14)	
High	33	24(13.87)	9(32.14)	

* Categorical variables analysis by the Chi-Squared Test or Fisher’s exact test and continuous variables analysis by the independent sample t-test

BMI: Body mass index; CCI: Charlson Comorbidity Index; Hb: hemoglobin; eGFR: estimated glomerular filtration rate; HTN: hypertension; DM: diabetes mellitus; R: Tumor size; E: Tumor location into the parenchyma; N: Nearness to the sinus or collecting system(mm); A: Anterior or Posterior; L: Location relative to the polar line; SD: Standard deviation

In Table 2, the analysis of perioperative, oncological, and functional outcomes was presented. There is no significant difference in operative time, surgical margin-free rate, recurrence rate, length of stay, postoperative changes in hemoglobin, estimated blood loss or complication rate between 2 groups. In the analysis of warm ischemic time (WIT), the distribution of patients receiving Off clamp or On clamp between Group A and Group B was not statistically significant. Within the On clamp group, Group B significantly experienced a prolonged WIT compared to Group A(18.90 vs. 22.60 min, *P* = 0.0486). Moreover, the significance of estimated blood loss(ml) was not observed between 2 groups (446.02 vs. 610.77 ml, *P* = 0.0778).

**Table 2:** Oncological and functional outcome after surgery

	Overall	Tumor size ≤ 7 cm		Tumor size > 7 cm	*P* value*
Number of patients, n	201	173	28		
Operation time, minute, mean(SD)	185.14(68.29)	180.37(64.077)	214.64(85.701)		0.0511
Warm ischemic, n(%)					0.6699
off clamp	42(20.9)	37(21.39)	5(17.86)		
on clamp	159(79.1)	136(78.61)	23(82.14)		
Ischemic time, minute, mean(SD)	19.43(8.33)	18.90(8.31)	22.60(7.92)		0.0486
Surgical margin, n(%)					1.0000
no free	5(2.49)	5(2.89)	0(0.00)		
free	196(97.51)	168(97.11)	28(100.00)		
Recurrence, n(%)					1.0000
no	194(96.52)	167(96.53)	27(96.43)		
yes	7(3.48)	6(3.47)	1(3.57)		
Complication, n(%)					0.0840
negative	193(96.02)	168(97.11)	25(89.29)		
positive	8(3.98)	5(2.89)	3(10.71)		
Trifecta achievement, n(%)					0.103
no	23(11.4)	17(9.8)	6(21.4)		
yes	178(88.6)	156(90.2)	22(78.6)		
Lengths of stay, day, mean(SD)	7.04(4.62)	6.97(4.83)	7.54 (3.04)		0.5461
Δ Hb, g/dL, mean(SD)	-1.76(1.20)	-1.74(1.20)	-1.88(1.21)		0.5411
Estimated blood loss, mL, mean(SD)	474.77(432.87)	446.02(420.09)	610.77(474.06)		0.0778
Δ eGFR, mL/min per 1.73 m2, mean(SD)					
POD1	-1.80(15.52)	-0.74(15.28)	-8.31(15.69)		0.0163
POM3	-4.57(12.57)	-3.92(12.59)	-8.55(11.92)		0.0709
POM6	-2.50(12.76)	-2.01(12.95)	-5.53(11.22)		0.1755
POY1	-3.51(12.67)	-3.00(12.81)	-6.68(11.46)		0.1537

* Categorical variables analysis by the Chi-Squared Test or Fisher’s exact test and continuous variables analysis by the independent sample t-test

Δ Hb: Change in hemoglobin; Δ eGFR: Change in estimated glomerular filtration rate; POD1: Postoperative day 1; POM3: Postoperative day 3; POM6: Postoperative day 6; POY1: Postoperative year 1; SD: Standard deviation

**Table 3:** eGFR changes over time in > 7 cm Tumors(*n* = 28)

Time Point	eGFR (mean ± SD)	𝚫eGFR(mean ± SD)	95% CI	*p*-value
Pre op (Baseline)	81.221 ± 15.3	NA	NA	NA
POD1	72.91 ± 19.04	-8.31 ± 15.69	[-0.746, 17.375]	0.092
POM3	72.68 ± 15.94	-8.55 ± 11.92	[1.661, 15.432]	0.008
POM6	75.69 ± 16.52	-5.53 ± 11.22	[-0.95, 12.015]	0.146
POY1	74.54 ± 17.12	-6.68 ± 11.46	[0.062, 13.303]	0.047

* Repeated measures ANOVA (Analysis of variance) was used to assess changes in eGFR over time

eGFR: Change in estimated glomerular filtration rate; Δ eGFR: Change in estimated glomerular filtration rate; POD1: Postoperative day 1; POM3: Postoperative day 3; POM6: Postoperative day 6; POY1: Postoperative year 1; SD: Standard deviation; 95 CI: 95% confidence interval

In the analysis of renal function, Group B exhibited higher mean declines in eGFR on POD1, POM3, POM6, and POY1 compared to Group A. However, statistical significance was merely observed in the decline of eGFR on POD1 (-0.74 vs. -8.31, *P* = 0.0163). No significant differences were noted in the decline of eGFR on POM3, POM6, or POY1 (-3.92 vs. -8.55, *P* = 0.0709; -2.01 vs. -5.53, *P* = 0.1755; and − 3.00 vs. -6.68, *P* = 0.1537, respectively). In the analysis for eGFR in > 7 cm tumors over time, the mean eGFR in Group B at baseline, POD1, POM3, POM6, and POY1 was 81.22, 72.19, 72.68, 75.69, and 74.54 mL/min/1.73 m², respectively. Significant declines were observed at POM3 (*p* = 0.008) and POY1 (*p* = 0.047), as illustrated in Fig. 2 and detailed in Table 3.

During the follow-up period, no mortality was observed among the 201 patients included in this study. Local recurrence occurred in 6 patients from Group A at 39.3, 43.8, 52.7, 58.6, 62.0, and 65.8 months postoperatively, and in 1 patient from Group B at 48.1 months postoperatively (3.47% vs. 3.57%, *P* = 1.0000). No distant metastasis was detected during the follow-up period.

Out of a total of 201 patients, 8 patients experienced complications, defined as greater than CDS grade 3. In Group A, 5 complications (2.89%) from 173 patients were noted and they were pseudoaneurysm managed by trans-arterial embolization (TAE), renal abscess requiring drainage by percutaneous nephrostomy, ureteropelvic junction injury and 2 urinary leakages requiring stent placement. Meanwhile, in Group B, 3 complications (10.71%) occurred from 28 patients. Among these 3, postoperative wound infection requiring surgical debridement, pseudoaneurysm requiring TAE and urinary leakage requiring stent placement were noted. However, in our analysis, these findings did not achieve statistical significance (5(2.89%) vs. 3(10.71%), *P* = 0.084).

In the assessment of trifecta, both groups showed favorable achievement rates in trifecta, with 90.2% in Group A and 78.6% in Group B (*p* = 0.103).

## Discussion

The advantage of PN to achieve comparable oncological outcome, renal function reservation, and complication rate versus RN has been mentioned in previous study [1, 5, 9, 10]. However, most of these studies focus on treating cT1 tumors. The discussion on the utilization of PN for cT2 tumors is relatively scarce. Additionally, due to the evolution of surgical approaches, from open to RAPN or LPN, the choice between transperitoneal PN or retroperitoneal PN has become another clinical issue [11, 12]. This study evaluates the feasibility and outcomes of retroperitoneal PN for renal tumors > 7 cm. In a cohort of 201 cases (100 LPN, 101 RAPN), there was no significant difference in complication rate, local recurrence rate, or trifecta achievement between the two groups, despite Group B presenting with higher RENAL scores and longer WIT. Focusing on eGFR changes over time in Group B, a significant declines were observed at POM3(-8.55) and POY1(-6.68), with tendency for partial recovery at POM6. Given this trend, clinicians should closely monitor eGFR decline beyond 6 months postoperatively, particularly in patients with tumors > 7 cm.

In comparison of functional outcome between PN and RN focusing on cT2 tumors, de Saint Aubert et al. conducted a retrospective study involving 130 patients with tumors > 7 cm (49 in the PN group and 81 in the RN group). A significant result of 8% versus 20% median eGFR reduction in PN group and RN group was noted [13]. These findings aligned with those reported by Breau et al., where a significant elevation of serum creatinine level was found in patients with cT2 tumors treated with RN compared to PN [14]. Some studies have further explored the risk factors for the development of chronic renal insufficiency. In terms of surgical approach, McKiernan et al. reported that patients undergoing RN had a greater risk of renal insufficiency compared to those undergoing PN [9]. Other identified risk factors included intermediate/high tumor complexity (e.g., RENAL score ≥ 7), solitary kidney, the on-clamp approach, and trifecta failure [15, 16]. Based on these studies, we can acknowledge the advantage of PN in renal function preservation. In the present study with 201 retroperitoneal PN cases, we also observed a favorable mean eGFR reduction of 3.51 in the overall cohort at the POY1 follow-up. A previous study also explored the association between tumor complexity and long-term eGFR reduction, showing that tumors with high complexity were associated with more prominent eGFR reduction at POY1 [17]. Although our Group B had a generally higher RENAL score, the eGFR reduction compared to Group A at POY1 was not significant.

The majority of our procedures utilized the on-clamp approach to control intraoperative bleeding and ensure a clear surgical field under laparoscopy. However, previous literature on robotic-assisted surgery has demonstrated the benefits of the off-clamp approach in preserving renal function without an increased complication rate or poorer oncological outcomes [18–20]. Despite treating tumors with a high RENAL score, a favorable trifecta could be achieved with the off-clamp approach compared with the on-clamp approach, and a prolonged WIT seemed to be a risk factor for trifecta failure and long-term eGFR reduction [18–20]. 

Regarding recurrence rates following PN, a study by Kim et al. compared the recurrence rates of patients with a RENAL score ≥ 10 after PN versus RN. They found no significant difference in the 5-year recurrence rate between the two groups [21]. As shown in Tables 1 and 2, with a mean follow-up of 41.87 months, 3.47% versus 3.57% for local recurrence rate in Group A and Group B were reported. There was no distant metastasis observed in our follow-up. The result supports that our retroperitoneal partial nephrectomy does not result in a higher recurrence rate, even when treating larger tumors.

For the analysis of complications, in a multicenter study reported by Porpiglia et al., although no difference in postoperative complication rates was found, transperitoneal PN exhibited a slightly higher intraoperative complication rate compared to retroperitoneal PN for cT1 tumors [11]. As for cT2 tumors, Matvey Tsivian et al. reported intraoperative and postoperative complication rates of 7.4% and 11% from 27 cases treated with transperitoneal LPN. The postoperative complications included a ureteral injury, a urinoma, and delayed bleeding [22]. In another study by J. Rouffilange et al., a total complication rate of 24.3% from 37 procedures was reported, with only 3 surgical complications (8.1%) ≥ CDS 3, including a urinary fistula, a deep abscess, and an early hernia requiring repairment. Our complication rate of 10.71% in treating cT2 tumors is similar to other studies. Besides, no gastrointestinal complication was noted in our experience with retroperitoneal PN, while increased gastrointestinal complications, including organ injuries or prolonged postoperative ileus, were observed to be associated with transperitoneal PN in previous studies [23]. 

Traditionally, trifecta assessment in PN has been based on negative surgical margins, WIT < 25 min, and the absence of CDS ≥ 3 complications, reflecting the common use of the on-clamp approach. However, emerging evidence suggests potential advantages of the off-clamp approach, particularly in preserving renal function. To better align with evolving surgical techniques, Brassetti et al. proposed a novel trifecta definition, incorporating: negative surgical margins, absence of CDS ≥ 3 complications, and postoperative eGFR decline < 30% [24]. Given that 20.9% of cases in our cohort used the off-clamp approach, we adopted this novel Trifecta criteria for analysis. The result shows that retroperitoneal PN achieves Trifecta rates of 90.2% in Group A and 78.6% in Group B.

In the literature review provided (Table 4), the approach to PN has evolved from an open to laparoscopic or robotic-assisted approach over generations [12]. Past studies have demonstrated comparable oncological outcomes, including recurrence and mortality rates between PN and RN under open surgery [5, 9, 11]. Despite considerations such as limited angles, surgical views, and risk of peritoneum injury, contemporary urologists still prefer laparoscopic and robotic-assisted surgeries to achieve minimal invasion.

**Table 4:** Review of series concerning NSS/PN for > 7 cm tumors

	Tumors(n)	Surgical approach	Tumor size (cm)	Tumor size range (cm)	Median follow-up (months)	Positive margins, n (%)	Major Complication(CDS > 3), n(%)	preoperative renal function	postoperative Renal function
Present study(2024)	28	R-PN: 28	9.90(mean)	7.2 ~ 19	47.5	0(0)	3(10.71)	(mean) eGFR:81.2 mL/min	(mean) - 6.68 mL/min
de Saint Aubert(2018)[13]	49	Open:48 LPN:1	8.6(mean)	NA	31	2(10.5)	8(16)	(mean) eGFR:76.9 mL/min	(mean) eGFR:68.9 mL/min
Rouffilange(2018) [25]	37	Open:34 T-PN:3	8 (median)	NA	31	NA	3(8.1)	(median) eGFR:80 mL/min	(median) eGFR:77 mL/min
Tsivian(2017) [22]	27	T-PN:27	8(median)	7 ~ 15	NA	1(3.7)	3(11.1)	NA	NA
Bigot(2014) [26]	168	Open:154 LPN:11 RAPN:3	8 (median)	7 ~ 18	30	14 (10.9)	33(19.6)	(mean) eGFR: 75.3mL/min	(mean) eGFR: 67.66 ml/min
Long(2012) [27]	49	RAPN:5	8.7(median)	7 ~ 30	13.1	5 (10.2)	6(12.2)	(median) Cr: 1.18 mg/dL	(median) Cr: 1.30 mg/dL
Becker(2011)[28]	91	Open: 90 T-PN:1	8(median)	7 ~ 17	28	NA	10(10.9)	median) eGFR:92.1 mL/min	(median) eGFR:81 mL/min
Karellas(2010) [29]	37	Open:32 LPN:5	7.5(median)	7–19	17	0	4(11)	(median) eGFR:65 mL/min	(median) eGFR:55 mL/min
Breau(2010) [14]	57	NA	7.5(median)	NA	38	NA	14(20.2)	(median) Cr.: 1.2 mg/dL	(median) + 9.5%
Jeldres(2009) [30]	29	NA	8.5(median)	NA	54	NA	NA	NA	NA
Hafez(1999) [2]	50	Open	9.9(median)	NA	47	NA	NA	NA	NA

CDS: Clavien-Dindo classification system; eGFR: Estimated glomerular filtration rate; Cr.: Creatinine; NSS: Nephron-sparing surgery; PN: Partial nephrectomy; R-PN: Retroperitoneal partial nephrectomy; T-PN: Transperitoneal partial nephrectomy; LPN: Laparoscopic partial nephrectomy; RAPN: Robotic-assisted partial nephrectomy; NA: Not applicable

There are several limitations in our study. First, as a retrospective, single-center study with a relatively small patient cohort, our findings may lack generalizability to larger populations. In particular, the cT2 group (*n* = 28) was significantly smaller than the cT1 group (*n* = 173), which may limit the ability to detect significant differences in outcomes. Second, while our functional analysis demonstrated a significant decline in eGFR in POY1, the information about renal function change with more extensive follow-up periods was unavailable. Likewise, while our study reported similar recurrence rates between two groups, longer follow-up is necessary to detect late recurrences. Besides, although the majority of our cases achieved negative surgical margins, detailed information including histopathological characteristics and margin width was lacking. Lastly, aside from tumor size, the preoperatively collected RENAL score showed a significant difference in the Exophytic/Endophytic parameter, which might introduce bias in patient selection as well as the surgical outcomes. Despite these limitations, due to the lack of existing research on partial nephrectomy for ≤ 7 cm versus > 7 cm renal tumors, we believe that our experience in retroperitoneal PN for treating renal tumors could provide a valuable reference to contemporary surgeons with regard to surgical techniques and patient selection. Finally, future studies with larger cohorts, longer follow-up, and more comprehensive oncological assessments are warranted to further address these limitations.

## Conclusion

In conclusion, despite preoperatively higher RENAL scores and perioperatively prolonged WIT, retroperitoneal PN for > 7 cm tumors could yield acceptable functional and oncological outcomes as well as a reasonable complication rate. Besides, no gastrointestinal complication was noted in our patients. Combined with previous studies suggesting renal function preservation with PN compared to RN, our findings support the feasibility of PN as a treatment option for patients with > 7 cm or intermediate/high complexity renal tumors through the retroperitoneal approach.

## Data Availability

All data generated or analyzed during this study are included in this published article.
